# The Changing Landscape of Genetic Risk: Age-Related Effects of Polygenic Scores for Suicidal Behaviors

**DOI:** 10.21203/rs.3.rs-10021308/v1

**Published:** 2026-09-17

**Authors:** Nathan Gillespie, Severine Lannoy, Mallory Stephenson, Robert Kirkpatrick, Zuriel Ceja, Miguel Renteria, Alexis C. Edwards

**Affiliations:** Virginia Commonwealth University; Virginia Commonwealth University; Virginia Commonwealth University; QIMR Berghofer Medical Research Institute; Virginia Commonwealth University

## Abstract

Suicidal thoughts and behaviors are a persistent public health challenge. Twin studies and genome-wide association studies (GWAS) have revealed moderate genetic influences. In exploratory analyses, we evaluated whether the impact of polygenic risk scores (PRSs) for suicide ideation (SI-PRS) and attempt (SA-PRS) varied across five waves of longitudinal data from the Add Health cohort, collected between 1994–1995 (Wave 1) and 2016–2018 (Wave 5), spanning ages 12 to 43 years. We used logistic regressions and Full-Information Maximum Likelihood structural equation modeling (SEM) in OpenMx to analyze associations between PRSs and measures of ideation and attempt while adjusting for sex, socioeconomic status, and ten ancestry principal components. Logistic regressions and the exploratory autoregressive SEM revealed modest but significant associations in later waves. Both approaches converged on the SI-PRS being associated with suicidal ideation at Wave 4 (ages 24–32) and the SA-PRS being associated with suicidal ideation at Wave 5 (ages 33–43). The SA-PRS was also associated with suicide attempts at Wave 5 in the SEM. There were no significant PRS-phenotype associations at Waves 1–3 (corresponding to adolescence and the transition to young adulthood). Our findings suggest that current PRSs, which are based primarily on adult GWAS samples, have stronger effect sizes during young adulthood and early mid-life rather than adolescence. Results highlight the need for expanded GWAS efforts that include age-stratified GWAS to capture developmental-specific genetic risks, which could better inform targeted prevention strategies.

## Introduction

Suicidal thoughts and behaviors represent a significant public health concern, with over 700,000 individuals dying by suicide worldwide annually, including approximately 49,000 in the US (1, 2). For every death by suicide, the WHO estimates that there are 20–25 attempts (1). Despite extensive research identifying and examining risk factors, our ability to predict and prevent these behaviors remains limited, highlighting the need for novel approaches to understanding suicide risk across development.

The heritability of suicidal thoughts and behaviors (STBs) has been established through twin and family studies, with estimates ranging from 0.17 to 0.55 across different outcomes (3–6). These findings indicate modest to moderate genetic influences on STBs risk, which provides a foundation for molecular genetic investigations. Recent advances in genomics have yielded promising results, with genome-wide association studies (GWAS) of suicide ideation, attempt, and death reporting SNP-based heritability estimates of h^2^SNP = 0.05, h^2^SNP = 0.07, and h^2^SNP = 0.16, respectively (7–9). These studies have also identified genetic loci and demonstrated that polygenic risk scores (PRSs) for suicide attempt are associated with STBs in independent samples (7, 10).

While these genomic advances represent important progress, most studies have examined genetic influences on suicide risk as static factors across the lifespan. Substantial evidence suggests that risk for STBs varies considerably across development, with distinct patterns of prevalence and manifestation from adolescence through adulthood. This heterogeneity raises the possibility that genetic risk factors have stronger associations at different life stages, as suggested by one Swedish family study (6). Identifying periods of heightened genetic vulnerability could help inform prevention efforts by revealing when genetic influences on STBs are strongest.

Longitudinal studies spanning adolescence to adulthood provide a unique opportunity to examine how the genetic risk for STBs interact with developmental processes. By applying the latest PRSs for suicide-related phenotypes to data collected across multiple time points across development, we can begin to identify whether certain ages or stages represent critical windows where the genetic liability is more strongly related to STB risk. The US National Longitudinal Study of Adolescent to Adult Health (Add Health) (11) is ideal for these types of investigations. It is a nationally representative cohort that enrolled > 20,000 adolescents (ages 12–18) in 1994–1995 and followed them over five waves into middle adulthood (Wave 5: ages 33–43). It also has repeated measures of suicidal thoughts and behaviors at each wave, as well as genome-wide genotyping performed at Wave 4.

### Aims

This study leveraged the most recent GWAS summary statistics for suicide ideation (9) and attempt (8) to calculate polygenic risk scores (PRSs) that we hypothesize would be predictive of suicide-related phenotypes. Specifically, we examined associations between PRSs for suicide ideation and attempt across five waves of longitudinal data spanning 22 years. Our aim was to determine whether or not genetic risks for suicidal behaviors show stronger associations during particular developmental periods, and also, to show if PRS effect sizes vary from adolescence through adulthood.

This approach extends previous research by applying recent genetic discoveries to a well-characterized longitudinal sample. It has the potential to identify developmental periods of heightened genetic vulnerability, which relevance for interventions.

## Materials/Subjects and Methods

### Participants and Study Design

Data were obtained from the National Longitudinal Study of Adolescent to Adult Health (Add Health) (11). The study followed participants over ~ 22 years. At Wave 1 (1994–1995), participants were in grades 7–12 (ages 12–18). Wave 2 data were collected in 1996 (age 13–19). Wave 3 data were collected in 2001–2002 (ages 18–26), Wave 4 in 2008–2009 (ages 24–32), and Wave 5 in 2016–2018 (ages 33–43). Note that data collection waves were spaced approximately 1, 5, 7, and 8 years apart, ensuring monotonic increases in participant age with no within-person overlap in age ranges across waves. Data collection at Waves 1 and 2 included both in-school and in-home components, with some Wave 1 data derived from parental reports, whereas Waves 3–5 primarily relied on in-home interviews. The sample was 46.4% female.

Approximately 50.3% of participants provided data at all five waves, with 34.6% contributing at four waves, 12.6% at three waves, 2.4% at two waves, and 0.1% at one wave. Attrition analyses indicated significant differential attrition by sex (χ^2^ = 442.12, df = 4, p < 0.001), with higher retention among males than females by Wave 5. Socioeconomic status (SES) also differed significantly between completers and non-completers, with 30.2% of completers classified as low SES compared to 36.7% of non-completers (χ^2^ test, p < 0.001), suggesting lower retention among participants with lower SES. No significant differences in attrition were observed based on baseline suicide ideation (χ^2^ = 0.32, df = 1, p > 0.05) or suicide attempt (χ^2^ = 0.00, df = 1, p > 0.05), indicating that missingness was unrelated to the primary phenotypes.

### Institutional Review Board (IRB) Statement and Informed Consent

All Add Health data collection, including the Wave 4 collection for genotyping, was approved by the Institutional Review Board of the University of North Carolina at Chapel Hill (IRB Study #05-1164 for Wave 4). All participants provided written informed consent in accordance with University of North Carolina School of Public Health IRB guidelines based on the U.S. Code of Federal Regulations on the Protection of Human Subjects (45CFR46).

### Measures & covariates

Suicide ideation and suicide attempt were assessed across all five waves. Ideation was assessed using the item, “*During the past 12 months, did you ever seriously think about committing suicide?*” with response options “no” (coded 0) and “yes” (coded 1). Attempt was assessed with, “*During the past 12 months, how many times did you actually attempt suicide?*” with a response of 0 being coded as 0, and any attempts being coded as 1. At Waves 1–3, the assessment of suicide attempt was entirely contingent upon endorsing suicide ideation, whereas at Waves 4–5, ideation was assessed independently.

To account for demographic factors, the models (see statistical analyses) included biological sex and SES as covariates. Only participants of empirically-derived European-like ancestry were included in the current analyses due to insufficient statistical power in other ancestry groups. An aggregate measure of SES was derived using items collected across waves that indicated whether the participant’s family had received public assistance, welfare payments, or food stamps. This variable was coded as binary, with 31.8% of participants classified as low SES.

### Genotyping, Quality Control (QC), and Calculating Polygenic Risk Scores (PRSs)

Genotyping and QC procedures are detailed elsewhere (12). Briefly, saliva samples were collected from 15,701 Add Health participants at Wave 4 using Oragene kits (13) and processed over three years. Genotyping was performed using Illumina Human Omni1-Quad and Omni-2.5 Quad BeadChips, including SNPs, ancestry-informative markers, and CNV probes from HapMap and the 1000 Genomes Project. QC involved SNP-level exclusions (call rates < 90%, minor allele frequency < 0.5%, triallelic variants, Hardy-Weinberg equilibrium failures stratified by ancestry) and sample-level exclusions (call rates < 90%, sex mismatches, heterozygosity outliers, duplicates with concordance < 0.99). After validation using HapMap controls and Exome Chip data, the genotyped sample included 9,974 participants.

PRSs for suicide attempt (SA-PRS) and suicide ideation (SI-PRS) were calculated using GWAS summary statistics for suicide ideation (9) and attempt (8). As described previously (14), we used PRS-CS (15) with the combined Human Genome Diversity Project and 1000 Genomes Project reference panel (16) to obtain weighted SNP effects across the whole spectrum of *p*-values. We applied standard SNP inclusion criteria (MAF > 0.5%, 90% variant and sample call rates, Hardy-Weinberg Equilibrium p > 10^−15^, and linkage disequilibrium pruning to r^2^ < 0.1–0.3; see the Supplement in Lannoy et al. (14) for full details), before calculating PRSs using Plink 2.0 (17). Finally, to account for potential population stratification, each PRS was residualized for the effects of the first 10 principal components (provided by Add Health to authorized users), before scaling each PRS to a mean of 0 and variance of 1 for all subsequent analyses.

### Statistical analysis

Statistical analyses were conducted using OpenMx (version 2.20.7) (18) in R (version 4.5.1 Patched; (19)), employing ordinal data analysis procedures to accommodate the binary nature of suicide ideation and attempt variables. Prior to structural equation modeling, pairwise correlations and their standard errors were estimated between suicidal ideation and attempt phenotypes (ordinal) and PRSs (continuous) across five waves using a custom-written *polypairwise* function in R with OpenMx. This function used Full Information Maximum Likelihood (FIML) to handle missing data, computing polychoric correlations for ordinal-ordinal pairs, polyserial correlations for ordinal-continuous pairs, and Pearson’s correlations for continuous-continuous pairs (see Supplement for source code).

#### Logistic regressions

At each of the five waves, cross-sectional logistic regressions were also performed to assess the impact of SI-PRS, SA-PRS, sex, and SES on the binary outcomes of suicidal ideation and attempts. Generalized linear models with a binomial distribution and logit link function were implemented in R (version 4.5.1). For each wave and outcome, the model regressed the phenotype on SI-PRS, SA-PRS, sex (coded as 1 = male, 2 = female), and SES (coded as described above). Odds ratios (ORs) and 95% confidence intervals (CIs) were calculated by exponentiating coefficients and CIs. To control for multiple comparisons across all regression results (5 waves × 2 outcomes, with 4 predictors per model), the raw *p*-values were adjusted using the Benjamini-Hochberg false discovery rate (FDR) method (20), with significant associations defined at FDR-adjusted p < 0.05.

#### Exploratory autoregression modeling

**Figure 1:**
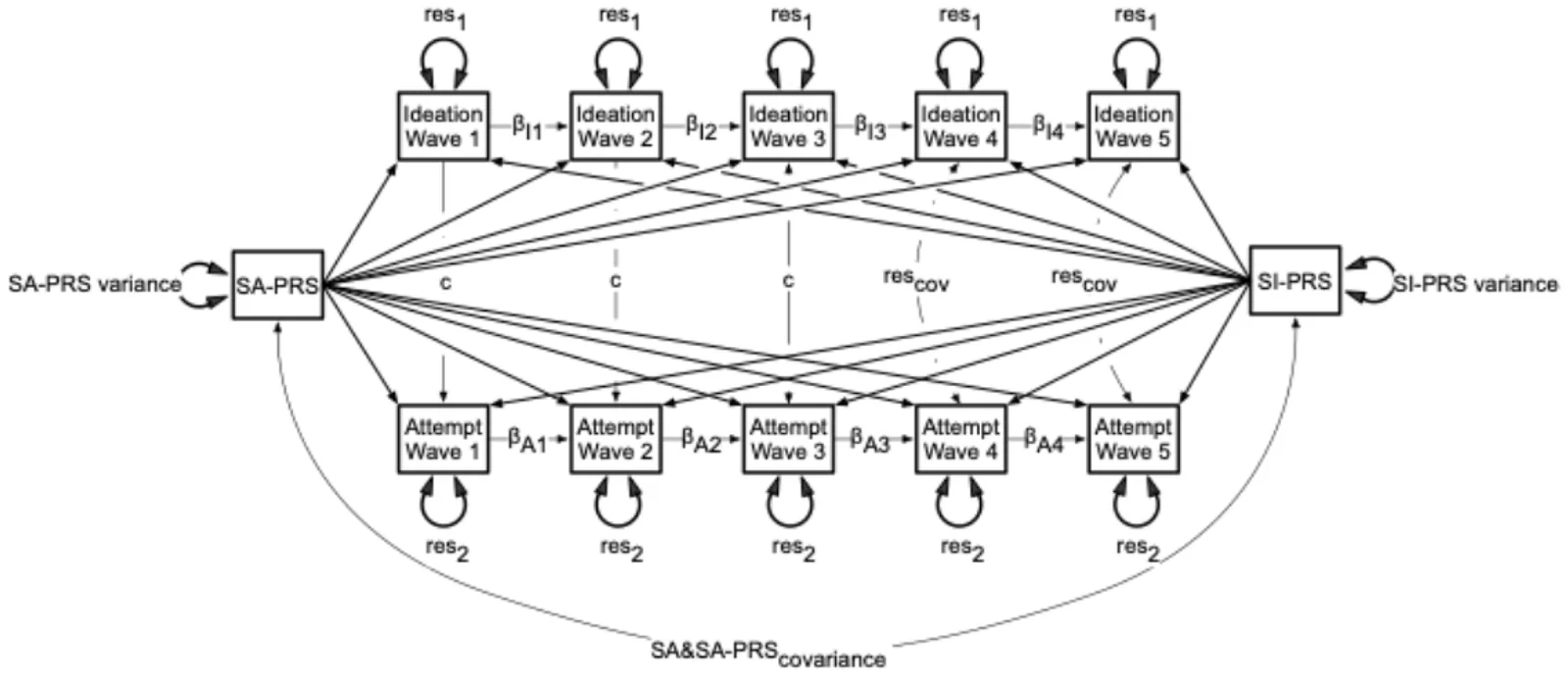
Note: Illustration of the bivariate autoregressive structural equation model for suicide ideation (top row) and suicide attempt (bottom row) across the five waves. Autoregressive paths connect successive measurements for both ideation and attempt. Residual variances were fixed to 1.0 for model identification. The model includes contingency pathways from ideation to attempt at Waves 1–3 (parameter c) to reflect the assessment protocol, and residual covariances between ideation and attempt at Waves 4–5 (parameter res_cov_). The suicide ideation PRS (SI-PRS) and suicide attempt PRS (SA-PRS) predict ideation and attempt at each wave, with these genetic effects allowed to vary freely across waves to capture any potential developmental differences. Covariates for sex, low socioeconomic status, and ancestry principal components were included as effects on the phenotypic means but are not shown for brevity. Bivariate Autoregressive Model of Suicide Ideation and Attempt Across Five Waves with Polygenic Risk Score Predictors

Next, we complemented the above wave-specific FDR-corrected logistic regressions, by fitting an exploratory bivariate autoregressive structural equation model (Fig. 1). This approach jointly models the contingencies between suicide ideation and attempt at Waves 1–3, the correlation between the two PRSs, and longitudinal autoregressive structure of the data.

The phenotypic covariance matrix revealed a simplex structure, with stronger correlations between adjacent waves, which weakened over longer intervals. We, therefore, fitted an autoregressive model to characterize the longitudinal relationships between suicidal ideation and attempt across the five waves. This model is well-suited for longitudinal data whenever observations at adjacent time points are more strongly correlated compared to those at larger time intervals.

For suicide ideation and attempt, the autoregressive paths were specified from each time point to the subsequent time point (e.g., Ideation Wave 1 → Ideation Wave 2, Ideation Wave 2 → Ideation Wave 3, etc.). We estimated a separate autoregressive coefficient for each transition (four parameters for ideation: β_I1_–β_I4_; four parameters for attempts: β_A1_–β_A4_). To account for the assessment protocols, we also included structural pathways. At Waves 1–3, because the suicide attempt items were only administered if participants endorsed suicide ideation, this contingency was modeled with directed paths (Ideation Wave 1 → Attempt Wave 1, Ideation Wave 2 → Attempt Wave 2, and Ideation Wave 3 → Attempt Wave 3), which we constrained to be equal (parameter ‘c’). At Waves 4–5, because attempts were assessed independently and the prevalence was low, the residual covariances between ideation and attempt were constrained to equality (parameter ‘rescov’).

The SI-PRS and SA-PRS were included as correlated predictors (parameter ‘SA- & SA-PRS_cov_’), with paths estimated freely to both the suicide ideation and attempt phenotypes at each wave to capture potential developmental differences in genetic effects (e.g., SA-PRS→Ideation Wave 1, SA-PRS→Ideation Wave 2, etc). The effects of sex and low SES were modeled as uniform influences on the means of all phenotypic variables via definition variables.

Finally, to ensure model identification and convergence, the residual variances of each measure of suicide ideation and attempt were fixed to 1.0. Fixing the residual variances to 1.0 sets the scale of the latent liability for each binary outcome and is standard practice when modelling ordinal data (21). Freely estimating the time-specific residuals would have been preferable. However, given the very sparse phenotypic data, the contingencies at Waves 1–3, and the large number of parameters, this approach resulted in non-convergence during preliminary model fitting attempts.

**Figure 2:**
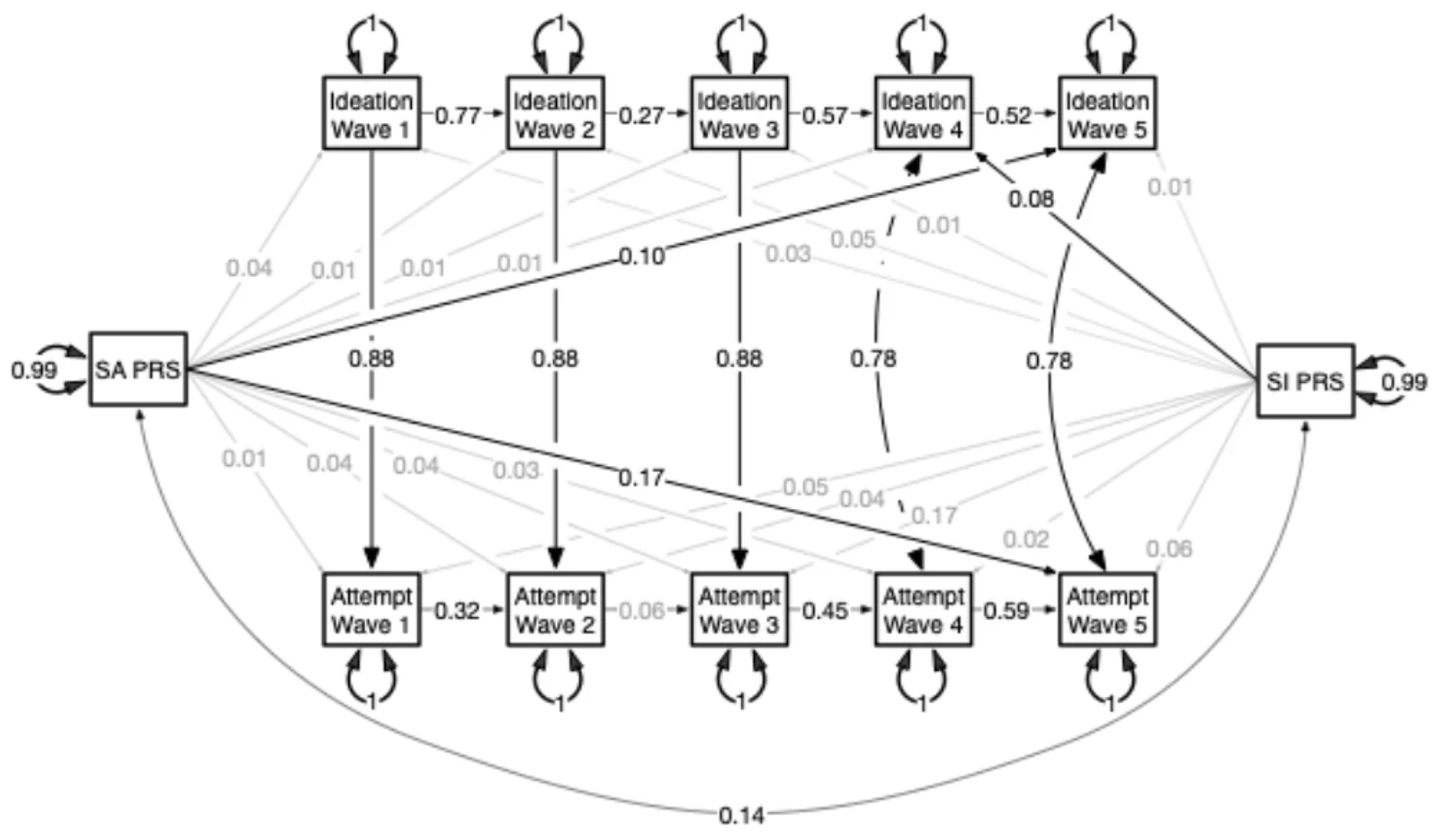
Bivariate Autoregressive Model of Suicide Ideation and Attempt Across Five Waves with Polygenic Risk Score Predictors And Parameter Estimates. Note: Non-significant parameters (p > 0.05) are displayed in light grey. FDR corrected *p*-values for all parameters, including effects of sex and low socioeconomic status (not shown for visual simplicity) are included in Supplementary Table S1.

We implemented this autoregressive model using a Reticular Action Model framework in OpenMx (Fig. 1). This model was fitted to the raw data using Full-Information Maximum Likelihood estimation with the SLSQP optimizer via mxTryHard in OpenMx. All parameter estimates and their corresponding *p*-values were based on the bivariate autoregressive structural equation model (illustrated in Figs. 1–2). We estimated *p*-values using z-tests (Beta estimates divided by their standard errors) with two-tailed probabilities based on the standard normal distribution. We then applied another Benjamini-Hochberg FDR correction (20) to all of the *p*-values associated with the covariance structure (not the means). Given the low number of suicide attempts at Waves 3–5 and the exploratory nature of this SEM analysis, our results should be interpreted with caution. The most reliable findings are those that appear in both this model and the FDR-corrected logistic regressions.

### Results

Table 1 presents the prevalence of suicide ideation and attempt across five waves of data collection spanning adolescence to adulthood. Suicide ideation was most prevalent at Waves 1 and 2 (15% and 13%, respectively, 12–19yrs), with lower rates observed at Waves 3–5 (7–8%, 18–43yrs). Among those endorsing ideation at Waves 1 and 2, the prevalence of suicide attempt was ~ 4%. When suicide attempt was assessed independently in all later waves, the prevalence was notably lower, with ~ 1–2% reporting an attempt between ages 18 and 43.

### Phenotypic Correlations

As shown in Table 2, the polygenic risk scores (PRSs) showed modest associations with suicidal ideation and attempt across waves. The full correlation matrix, including all phenotypic inter-correlations and standard errors, is presented in Supplementary Table S1. The within-wave polychoric correlations between suicide ideation and attempt were strongest in the later waves (r = 0.77 at Waves 4 and 5). Note that the within-wave correlations at Waves 1–3 were undefined due to the contingent assessment of attempts on ideation.

The cross-wave correlations within suicidal ideation ranged from 0.21 to 0.60, indicating moderate temporal stability. With the exception of the (smallest) correlation between ideation at waves 2 and 3 (r = 0.21), the near-adjacent waves generally showed the highest correlations (e.g., Waves 1–2: r = 0.60; Waves 3–4: r = 0.45; Waves 4–5: r = 0.51), whereas the correlation over the longest interval (Waves 1–5: r = 0.31) was modest.

The cross-wave correlations within suicide attempts ranged from 0.04 to 0.52 but showed a highly similar pattern: stronger associations between adjacent assessments (e.g., Waves 1–2: r = 0.52; Waves 4–5: r = 0.46), with the exception of a markedly lower correlation between Waves 2 and 3 (r = 0.16). Over longer intervals, such as between Waves 1–5 (r = 0.28) and Waves 2–5 (r = 0.34), some degree of temporal stability is evident.

Cross-trait cross-wave correlations were also noteworthy. For instance, Wave 1 ideation also correlated with attempts at Waves 2 (r = 0.18), 4 (r = 0.20) and 5 (r = 0.18). Similarly, Wave 2 ideation correlated with attempts at Waves 4 (r = 0.27) and 5 (r = 0.23), while Wave 3 ideation correlated with attempts at Waves 4 (r = 0.38) and 5 (r = 0.28). Additionally, Wave 4 ideation correlated with Wave 5 attempts (r = 0.50). In contrast, the correlations between Wave 1 attempt and Wave 5 ideation (r = 0.04) and between Wave 2 attempt and Wave 5 ideation (r = 0.15) were much smaller, as were those between Wave 3 attempt and Wave 4 ideation (r = 0.20) and between Wave 4 attempt and Wave 5 ideation (r = 0.34).

The polyserial correlations between the SI- PRS and suicidal ideation at each wave and ranged from r = 0.03 to 0.09. Correlations between suicide attempts at each wave and the SA-PRS were smaller in magnitude (r = −0.04 to 0.05). Notwithstanding the large standard errors, the highest phenotype-PRS correlations were observed between Wave 3 attempt and the SI-PRS (r = 0.13), as well as between Wave 5 attempt and the SI-PRS (r = 0.13). The correlation between the suicidal ideation and attempt PRSs was small (r = 0.14), suggesting limited genetic overlap.

Regarding the covariates, sex (where 1 = male, 2 = female) revealed positive correlations with suicidal ideation in earlier waves (r = 0.15 to 0.18 at Waves 1–2) but near-zero to very small negative associations in later waves (r = − 0.02 to 0.01 at Waves 3–5), indicating some degree of developmental variation across time. For suicide attempts, positive correlations with sex were observed across most waves (r = 0.02 to 0.31), with the strongest in adolescence—most notably with Wave 2 attempts (r = 0.31).

Lower SES showed consistently positive associations with suicidal ideation across all five waves (r = 0.09–0.20), with values of 0.09–0.18 in Waves 1–3 and 0.17–0.20 in Waves 4–5. Associations with suicide attempts were also positive but more variable (r = 0.05 to 0.24). Overall, all correlations were small to moderate in size, suggesting that these demographic factors exerted modest but meaningful influences on the two suicide-related outcomes.

### Logistic regressions

Logistic regression analyses revealed limited but noteworthy associations between the suicidal phenotypes and the PRSs (Table 3). Full results including all covariates, z-values, and raw p-values are presented in Supplementary Table S2. The suicide attempt PRS (SA-PRS) was significantly associated with increased odds of suicidal ideation at Wave 5 (OR = 1.18), whereas the suicide ideation PRS (SI-PRS) was linked to higher odds of ideation at Wave 4 (OR = 1.18). Neither PRS was significantly associated with ideation or attempts at Waves 1–3. Among the covariates, sex showed consistent associations with ideation at Waves 1 (OR = 1.34) and 2 (OR = 1.43), and with attempts at Waves 1 (OR = 1.49) and 2 (OR = 1.76), indicating higher risk among females. Lower SES was also positively associated with ideation at Waves 1 (OR = 1.22), 2 (OR = 1.40), 4 (OR = 1.47), and 5 (OR = 1.55), and with attempts at Waves 4 (OR = 1.69) and 5 (OR = 1.79).

### Autoregression modelling

We next modelled the associations between the PRSs for suicidal ideation and attempt, and the phenotypic measures across the five waves using a structural equation model. This model simultaneously adjusted for the effects of sex and SES by including them as definition variables using single, time-invariant regression coefficients on all ten suicidal phenotypes. Full parameter estimates and *p*-values for all model parameters are provided in Supplementary Table S3.

As illustrated in Fig. 2, the SI-PRS and SA-PRS were allowed to covary (cov = 0.14, p < .0001), with both PRSs regressed onto all ten suicidal phenotypes. At Waves 1–3, suicide attempt was contingent upon ideation, as illustrated by the pathway coefficient (c = 0.88, p < 0.0001). In contrast, the residual covariances between ideation and attempt were estimated at Waves 4–5 (res_cov_ = 0.78, p < 0.0001). The autoregressive pathways showed only moderate stability for suicidal ideation (ranging from 0.27 to 0.77) and more variability for suicide attempts (ranging from 0.06 to 0.59).

After adjustment for sex, SES, the contingencies at Waves 1–3, and the covariance between the two PRSs, only three PRS effects remained statistically significant after FDR correction: (i) the SI-PRS was significantly associated with suicidal ideation at Wave 4 (β = 0.08, p < 0.01); (ii) the SA-PRS was significantly associated with suicidal ideation at Wave 5 (β = 0.10, p < 0.0001); and (iii) the SA-PRS was significantly associated with suicide attempts at Wave 5 (β = 0.17, p < 0.001). Being female (β = 0.08, p < 0.0001) and reporting low SES (β = 0.17, p < 0.0001) were associated with higher mean liabilities across all suicidal phenotypes.

To facilitate interpretation of the model coefficients in Fig. 2, Table 4 shows the standardized correlations implied by the exploratory SEM. These values are analogous to zero-order polychoric correlations and reflect associations after adjusting for sex, SES, autoregressive stability, within-wave contingencies (Waves 1–3), residual covariances (Waves 4–5), and the correlation between the two PRSs.

For the three PRS effects that remained statistically significant after FDR correction, the model-implied correlations for these pathways were modest (SI-PRS to Wave 4 ideation: r = 0.08; SA-PRS to Wave 5 ideation: r = 0.09; SA-PRS to Wave 5 attempt: r = 0.15). We note, also, that the largest pathway coefficient in Fig. 2 from any PRS to a suicidal phenotype (SI-PRS to suicide attempt at Wave 3, β = 0.17) did not remain statistically significant after FDR correction (p = 0.06), even though the corresponding model-implied correlation was one of the largest (r = 0.13). All other model-implied correlations between the PRSs and the suicidal ideation and attempt variables ranged from − 0.01 to 0.10.

## Discussion

Our study leveraged GWAS summary statistics to compute PRSs for suicide ideation (SI-PRS) and suicide attempt (SA-PRS), before examining their association with suicide ideation and attempts across five waves of longitudinal data that spanned adolescence to adulthood in the Add Health cohort. Our findings, based on cross-sectional logistic regressions and the exploratory autoregressive SEM, revealed limited but significant associations mostly in later developmental stages. The SI-PRS was significantly associated with suicidal ideation at Wave 4 (ages 24–32) and the SA-PRS was significantly associated with suicidal ideation at Wave 5 (ages 33–43) in both approaches. The SA-PRS was also associated with suicide attempts at Wave 5 in the SEM (but not in the cross-sectional logistic regressions after FDR correction). These results suggest that measured genetic risks have stronger associations at later waves (corresponding to young adulthood and early mid-life) compared to earlier waves (corresponding to adolescence and the transition to young adulthood). Correlations and cross-sectional logistic regressions also supported only modest temporal stability in suicidal phenotypes and small genetic overlaps. Our results suggest that there are developmental periods during which the currently available measured genetic risks for ideation and attempt have stronger effects. Consequently, PRSs are more likely to identify risk during young adulthood and early mid-life than during adolescence. These periods could point to important windows of heightened genetic vulnerability. However, formal tests of age moderation or mechanistic explanations were not conducted.

Previous reports have demonstrated that PRSs for suicide ideation and attempt are associated with STBs phenotypes in young adulthood and adulthood, consistent with the age ranges observed in our significant findings for SI-PRS at Wave 4 (ages 24–30) and SA-PRS at Wave 5 (ages 33–43) (22, 23). This is unsurprising given that the discovery GWAS samples (8, 9) mostly comprised of adults, including large numbers of Million Veteran Program (MVP) participants. GWAS based heavily on military-ascertained samples can raise concerns about generalizability. However, Docherty et al. (2023) reported highly comparable GWAS signals across their fixed-effects and meta-regression results, which suggests that civilian versus military ascertainment was unlikely to confound the observed GWAS signals. Nevertheless, the mostly adult composition of these GWAS samples could still limit their sensitivity to adolescent-specific genetic influences. This underscores a critical need for discovery GWAS to include more adolescents to better capture genetic vulnerabilities relevant to earlier developmental periods. This could enhance the predictive power of PRSs across the lifespan and inform more effective, age-specific interventions. Future analyses should also consider evaluating whether the impact of genetic variants differs as a function of age, similar to analyses that have applied such an approach in the context of alcohol use frequency (24) or to interactions between genetic factors and measured environmental exposures (25).

This need for targeted GWAS discovery samples also underscores the importance of investments in genotyping, as well as additional phenotyping and genotyping of ancestrally diverse populations, along with larger samples to enhance the quality and statistical power of GWAS summary statistics. Improved genomic data will expand the relevance of PRS, which currently fall short of clinical utility for outcomes related to suicidality. Such advancements are critical to better leveraging genetic information for identifying and intervening in at-risk populations.

While the role of genetic influences across development was the focus of our analyses, findings from the phenotypic component are also notable. The autoregressive paths underscore the temporally dynamic nature of suicide-related phenotypes. For example, the path from Waves 1 to Wave 2 ideation was strong (β = 0.77), but declined considerably from Waves 2–3 (β = 0.27), potentially reflecting the years-long lag between assessments. Similarly, the autoregressive path between attempt between Waves 2 and 3 was not statistically significant. However, stability between Waves 3–5 was moderate: This could be a function of behavioral consistency once one reaches adulthood. A prior study reported quantitative and qualitative differences in the impact of genetic factors during a similar time frame, which in conjunction with low environmental correlations across time could explain the current observations (6). Previous research supports the notion that the experience of suicidality is inconsistent across the life course (26, 27), e.g., in the form of adolescent-limited suicidality. We note, though, that Add Health’s assessment of ideation/attempts within the past 12 months (rather than since the previous report) makes it difficult to determine what occurred during the intervening years between assessments. Nevertheless, this temporal inconsistency has implications for risk assessment, in that previous suicidality might be a less effective predictor of future ideation/attempts when spanning adolescence to adulthood.

We further note the assessment structure for Waves 4 and 5 – i.e., asking participants about past-year attempts regardless of their endorsement of ideation – showed that some individuals engaged in suicidal behaviors during periods in which they did not report ideation. This challenges assumptions that attempt is contingent on recent ideation and suggests that ideation-to-action theoretical frameworks (28, 29) are unlikely to capture all pathways to suicide attempt. This finding aligns with our prior multivariate twin study in young adults, which revealed largely overlapping genetic risks across suicidal ideation, planning, and attempt (rA = 0.85–0.99), but only moderate non-shared environmental correlations (30). One implication of this finding is that suicidality assessments, whether administered for clinical or research purposes, would benefit from addressing attempt even in the absence of ideation endorsement.

### Limitations

The findings of this study should be interpreted in the context of six potential limitations.

First, the discovery GWAS samples used to compute the PRSs for suicide ideation and attempt were relatively underpowered compared to GWAS for other complex psychiatric traits. This limits the accuracy of the PRSs, while underscoring the need for ongoing international consortia efforts aimed at improving discovery GWAS, which would yield more accurate beta weights and consequently enhance the applicability of PRSs.

Second, the limited and non-significant path estimates of the PRSs for ideation and attempt when predicting the phenotypes at Waves 1–2 (adolescent periods) are consistent with the discovery GWAS samples being largely comprised of adult participants. At the same time, the discovery GWAS consortia typically adjust for age, which can obscure any age-related genetic effects. By adjusting for age, these analyses were likely to identify age-invariant genetic associations, which can miss developmental stage-specific risks. Future studies should employ age-stratified analyses or model age-dependent effects to better capture such specificity.

Third, the small number of suicide attempts at Wave 5 likely reduced the statistical power for PRS associations. Findings at this wave should be interpreted with caution. To preserve statistical power, our SEM analysis combined data across males and females. This approach may conceal sex-specific genetic influences on STBs. As larger samples become available, sex-stratified analyses could also reveal distinct developmental patterns of genetic risk.

Fourth, our study sample was predominantly of European ancestry, limiting the generalizability of findings to populations with different or admixed ancestral backgrounds.

Fifth, an independent replication sample was unavailable. However, the longitudinal design of our study provided an internal replication across developmental periods, which strengthens the confidence in the observed patterns.

Sixth, the phenotypic measures were analyzed according to the wave-based structure of the Add Health study design, rather than non-overlapping age-based intervals. Although the Wave 2 suicide attempt included 20 participants aged exactly 13 years (3.8% of the analytic sample), we retained the wave-based analyses to remain consistent with the data-collection protocol. Finer-grained age bands might have allowed for more precise PRS-phenotype associations. However, we judged that this was would have had a minimal impact on the overall pattern, and, therefore, kept the wave-based structure.

Additional limitations could include self-report biases that can vary by developmental stage, attrition by sex and SES, or gene-environment interactions across development. Future research that includes additional measured environments or diverse - omics data has the potential to offer more insights into the development of suicidal behaviors.

## Conclusion

In a nationally representative sample spanning ages 12 to 43 years, we found that genetic influences on suicidal thoughts and behaviors became stronger in young adulthood and middle adulthood (Waves 4 and 5). When combined with future, age-specific genetic discoveries, our findings may improve our understanding of the biology of suicidality, and ultimately, help to guide risk assessments and targeted preventions.

## Supplementary Material

This is a list of supplementary files associated with this preprint. Click to download.

- SupplementaryTables.xlsx
- SupplementaryTables.xlsx

Supplementary information is available at Molecular Psychiatry’s website.

## Figures and Tables

**Table 1:** Prevalence of Suicidal Ideation and Attempt Across Five Waves of the Add Health Study.

Wave & Age Range	Sample	Suicide Ideation	Suicide Attempt
Wave 1 (12-18yrs) Adolescence	5,062	766 (15.1%)	204 (4.0%)
Wave 2 (13-19yrs) Adolescence	3,812	497 (13.0%)	148 (3.9%)
Wave 3 (18-24yrs) Young Adulthood	4,246	317 (7.5%)	67 (1.6%)
Wave 4 (24-30yrs) Young Adulthood	5,081	397 (7.8%)	82 (1.6%)
Wave 5 (33-43yrs) Adulthood	3,849	270 (7.0%)	46 (1.2%)

**Table 2:** Pairwise Polychoric Correlations (with Standard Errors) Between Polygenic Risk Scores and Suicidal Ideation and Attempt Across Five Waves.

PRS	Wave 1		Wave 2		Wave 3		Wave 4		Wave 5	
	Ideation	Attempt	Ideation	Attempt	Ideation	Attempt	Ideation	Attempt	Ideation	Attempt
Suicide Ideation PRS	0.04 (0.02)	0.06 (0.05)	0.06 (0.03)	0.05 (0.06)	0.03 (0.03)	0.13 (0.07)	0.09 (0.03)	0.08 (0.04)	0.07 (0.03)	0.13 (0.06)
Suicide Attempt PRS	0.05 (0.02)	−0.04 (0.03)	0.04 (0.02)	−0.02 (0.03)	0.02 (0.02)	−0.01 (0.04)	0.04 (0.03)	0.05 (0.04)	0.04 (0.01)	0.02 (0.01)

**Note**: The correlation between SI-PRS and SA-PRS was 0.14 (SE = 0.02). Full correlation matrix is presented in Supplementary Table S1.

**Table 3:** Logistic Regression Associations of Polygenic Risk Scores with Suicidal Ideation and Attempt Across Five Waves (FDR-adjusted).

Outcome / Wave	Predictor	β (SE)	OR (95%CIs)	FDR q-value
**Suicide Ideation**	Wave 1 (12-18yrs)	SI PRS	0.05 (0.04)	1.05 (0.97, 1.14)	0.3442
		SA PRS	0.08 (0.04)	1.08 (1.00, 1.17)	0.1280
	Wave 2 (13-19yrs)	SI PRS	0.08 (0.05)	1.08 (0.98, 1.20)	0.2256
		SA PRS	0.03 (0.05)	1.03 (0.94, 1.14)	0.6780
	Wave 3 (18-24yrs	SI PRS	0.05 (0.06)	1.05 (0.93, 1.18)	0.6123
		SA PRS	0.01 (0.06)	1.01 (0.90, 1.14)	0.9224
	Wave 4 (24-3yrs)	SI PRS	0.17 (0.05)	1.18 (1.06, 1.32)	**0.0080**
		SA PRS	0.02 (0.05)	1.02 (0.92, 1.13)	0.9218
	Wave 5 (33-43yrs)	SI PRS	0.10 (0.07)	1.11 (0.97, 1.26)	0.2535
		SA PRS	0.17 (0.06)	1.18 (1.04, 1.34)	**0.0320**
**Suicide Attempt**	Wave 1 (12-18yrs)	SI PRS	0.09 (0.08)	1.09 (0.93, 1.29)	0.4595
		SA PRS	0.02 (0.08)	1.02 (0.87, 1.21)	0.9218
	Wave 2 (13-19yrs)	SI PRS	0.07 (0.11)	1.07 (0.87, 1.32)	0.6780
		SA PRS	0.03 (0.10)	1.03 (0.84, 1.25)	0.9218
	Wave 3 (18-24yrs)	SI PRS	0.23 (0.13)	1.26 (0.97, 1.64)	0.2047
		SA PRS	−0.02 (0.15)	0.98 (0.74, 1.30)	0.9224
	Wave 4 (24-3yrs)	SI PRS	0.15 (0.11)	1.16 (0.93, 1.45)	0.3320
		SA PRS	0.02 (0.11)	1.02 (0.82, 1.27)	0.9224
	Wave 5 (33-43yrs)	SI PRS	0.27 (0.16)	1.31 (0.96, 1.80)	0.2047
		SA PRS	0.29 (0.15)	1.33 (0.98, 1.80)	0.1731

**Table 4:** Model-Implied Correlations from the Bivariate Autoregressive Structural Equation Model (Adjusted for Sex and Socioeconomic Status).

Measure	1.	2.	3.	4.	5.	6.	7.	8.	9.	10.	11.	12.
1. Wave 1 Ideation	1.00											
2. Wave 1 Attempt	0.66	1.00										
3. Wave 2 Ideation	0.61	0.41	1.00									
4. Wave 2 Attempt	0.57	0.52	0.77	1.00								
5. Wave 3 Ideation	0.20	0.13	0.33	0.25	1.00							
6. Wave 3 Attempt	0.18	0.13	0.28	0.24	0.69	1.00						
7. Wave 4 Ideation	0.11	0.07	0.17	0.13	0.52	0.36	1.00					
8. Wave 4 Attempt	0.09	0.07	0.15	0.13	0.37	0.53	0.75	1.00				
9. Wave 5 Ideation	0.06	0.04	0.09	0.07	0.27	0.19	0.52	0.39	1.00			
10. Wave 5 Attempt	0.06	0.05	0.09	0.08	0.21	0.30	0.43	0.57	0.77	1.00		
11. Suicide Ideation PRS	0.03	0.06	0.06	0.07	0.02	0.13	0.08	0.06	0.06	0.10	1.00	
12. Suicide Attempt PRS	0.04	0.04	0.02	0.01	0.00	−0.01	0.02	0.01	0.09	0.15	0.14	1.00

**Note**: Model-implied correlations represent the pairwise associations between suicide ideation and attempt phenotypes, and the two PRSs (SI-PRS and SA-PRS) as predicted by the bivariate autoregressive structural equation model. These correlations are derived from the model’s estimated parameters—including autoregressive paths, cross-sectional contingency pathways at Waves 1–3, residual covariances at Waves 4–5, and the direct effects from the SI-PRS and SA-PRS —after adjusting for the effects of sex and low SES on the phenotypic means. Specifically, sex and low SES were modeled as having uniform effects on the underlying latent liability means of all ideation and attempt variables. By incorporating these covariates, the model-implied correlations reflect associations between the phenotypes and the PRSs that are independent of the variances attributable to sex and SES. These correlations, therefore, provide a clearer view of longitudinal stability, cross-phenotype contingencies, and the genetic associations free from demographic confounding.

## Data Availability

The Add Health data used in this study are available through the Carolina Population Center at the University of North Carolina at Chapel Hill. Access requires an approved application and data use agreement. GWAS summary statistics for suicide ideation and attempt are publicly available from the original publications (8, 9).
